# Supplementary material for: Decreased Prosaposin and Progranulin in the Cingulate Cortex Are Associated with Schizophrenia Pathophysiology
Source: Int J Mol Sci. 2022 Oct 10;23(19):12056. doi: 10.3390/ijms231912056 (PMC9570388; doi:10.3390/ijms231912056)
Supplement: Supplementary file 1 [file ijms-23-12056-s001.zip › ijms-1914987-supplementary.pdf]

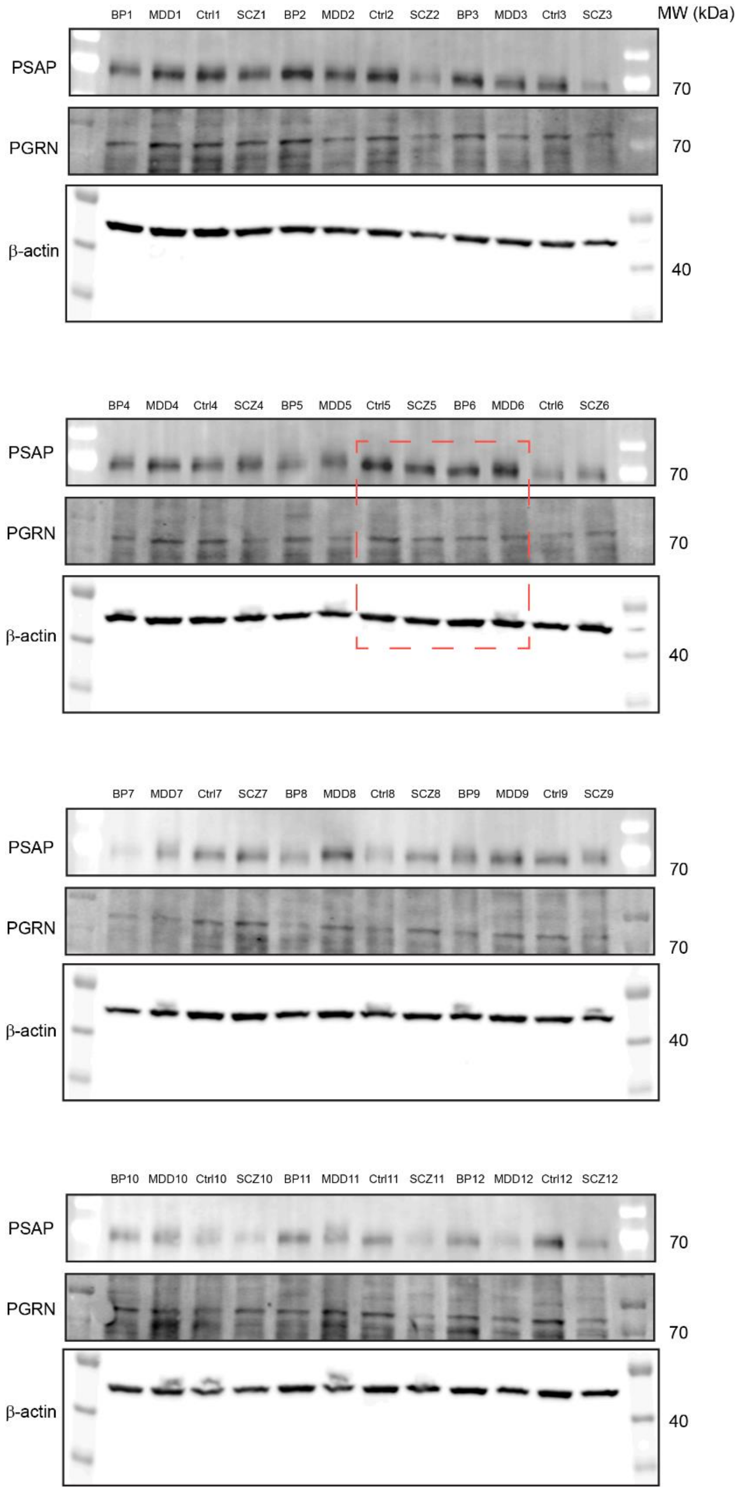

Supplementary Figure S1. Uncropped original blots for Figure 1. Red box indicates representative bands shown in Figure 1. Ctrl, healthy control; SCZ, schizophrenia; BP, bipolar disorders; MDD, major depressive disorders. MW, molecular weight.
